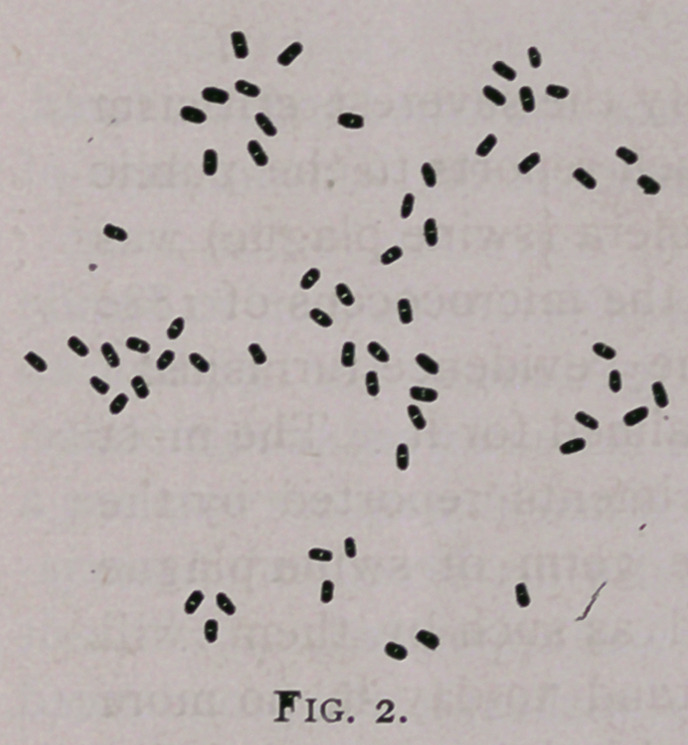# The Etiology of Southern Cattle Plague—Texas Fever

**Published:** 1892-07

**Authors:** Frank S. Billings

**Affiliations:** Director of the Patho-Biological Laboratory of the State University of Nebraska


THE JOURNAL
OF
COMPARATIVE MEDICINE AND
VETERINARY ARCHIVES.

VOL. XIII. JULY, 1892. NO. 7.

### THE ETIOLOGY OF SOUTHERN CATTLE PLAGUE—TEXAS FEVER.

BY FRANK S. BILLINGS, Director of the Patho-Biological Laboratory of the State University of Nebraska.

In the year 1888 I published my first investigations in the etiology of this disease, and in 1889 a second edition was published under the title of "Original Investigations in Cattle Diseases in Nebraska," those on the "Corn Stalk Disease, Hydrophobia in Cattle," and on infectious disease of the external sexual organs, and the cornea in cattle having been added. As has thus far been invariably the case, the Agricultural Department in Washington soon after began to throw doubts on the reliability of these investigations, so far as the announcement of a specific-micro-organism had been made, and in the case of Southern cattle plague to assert the existence of an entirely different organism as the cause of that disease. This was done absolutely on unscientific grounds and utterly without proof. With regard to the "corn stalk disease," a member of the department had also said, "*I do not take very much stock in the germ theory of this disease, because to accept that we must throw aside pretty much of our experience.*" As the etiology of that disease is now established beyond question, supported as it is by the work of Nocard in France, and Bowhill in England, it is evident that the "stock," which the farmers of the United States and scientific investigators should take in the announcements on the etiology of specific diseases, from the National Agricultural Department at Washington, should fall very much "below par." In reality, it must always be taken *cum grano salis.*

That same individual has also said of the Nebraska investigations, "Billings has boldly announced a great many discoveries that have not fulfilled the expectations of his friends." (Reply to Dr. Peters, JOURNAL OF COMP. MED., Vol. XIII, p. 35, 1892.) *Let me again, with equal boldness, announce my utmost confidence that all my discoveries will eventually fulfill the most exacting expectations of my friends, and equally disappoint those who are not, even to inoculation against swine plague.*

I am only too well aware that the unfortunate polemical discussion which circumstances have forced me into, has essentially militated against the acceptance of my work, and often prevented an unbiased appreciation of it by other investigators as well as many intelligent readers in the lay public. Still, the general result has been perfectly satisfactory to me as well as those most directly interested, the Stock Breeders of Nebraska, as is evinced by their insisting on my recall here and my retention in the face of the most bitter and determined opposition of the National Agricultural Department. I was fully aware that my true purpose in this polemic would be misunderstood. I knew full well that European investigators would be a long time in coming to an appreciation of the fact, that dishonest and unreliable work could possibly be put on the public by a department of government. I have in my possession scores of letters from among the ablest men in Europe, asking me, "Is it possible that such things can be?" "Was I sure that I was not mistaken in the matter?" They repeatedly said, "that such things must be impossible; that no investigator, who would do as I claimed, would be retained an hour in the employ of any European government;" which is true.

While European governments undoubtedly are still very imperfect organizations, politics in those countries have not yet degenerated to the machine-rottenness and corruption which is fast threatening republican institutions in this country with disruption or the land with dangerous revolution. We cannot go on in our present course many years longer without one or the other occurring. The despotism of these rotten-machine politics is fast driving reflecting and honest minded citizens to a condition verging on desperation, without regard to their fortunate or unfortunate position in the social scale. These forces will eventually unite, and then look out. No man can foretell what the result may be. The people are honest at the bottom, though much enslaved at present. Republican institutions are much slower in their movements than rebellion under monarchial governments, but the resulting revolution is liable to be much more radical and far reaching in its effects. I have thus plainly stated my opinions in order to show my readers the true spirit which has actuated me in my attack on the work of the Department of Agriculture. To those who know me, to my professors and colleagues in Germany, who know that from the day I began to study, *my ambition has not been to gather honors as a discoverer in the realm of investigation in the branch of science in which I have worked, but rather to do the best I could, in order to demonstrate the value of scientific investigation to the people of this country, that it might be inaugurated and supported by them through their governments, National and State.*

It is well known, that before I came to Nebraska, I had given fully as much, if not more, attention to the diseases of human life than of animal, and that it was my earnest hope to have given my entire energies to the study of the infectious diseases of child life. In that I completely failed. Neither the intelligence of any representative body nor the public spirit of any of our citizens were capable of being aroused to any enthusiasm in that direction. Necessity, in one form or another, has been the stimulus to every advance made by the race in its historic evolution. Necessity, the great losses in live stock from disease, was the cause of my call and recall to Nebraska. The necessity of saving the "Almighty Dollar" has thus far been the only stimulus to the investigation of infectious diseases in this country. The necessity of saving a human life from the miseries of disease has not yet made itself apparent to the American people. When it does the work will be inaugurated and well started. To educate the people in this direction has been the stimulus to, and earnest purpose of my own work. To attain this end the best I could do was necessary. I am absolutely without ambition for priority as a discoverer, as I am fortunately beyond the necessity of looking to my position for daily support. If I am a "crank" on any one thing, it is for honesty, to a fanatical degree, in the public service. Though a Democrat of Democrats I know no party, and am absolutely free from party affiliations. "*The good of the people first, the good of the people last, and the good of the people every time,*" is the one and only principle which I lay any claim to in my actions. With such ideas anyone can readily see what must have been the sentiments and results when I found the reports of the Agricultural Department, from the year 1880 to 1885, to be absolutely and unequivocally false; to be a tissue of impossibilities so far as they had reference to the specific cause of swine plague, with the exception of the investigations of Dr. J. Detmers, which were claimed to be wrong by the Government itself, but were reasonably correct.

With an effrontery that could only come from one hardened with the brazen corruption of an American politician, the Agricultural Department at Washington now tells the people of this country, and defies any high moral character they may have, that the germ of hog cholera (swine plague) was not discovered until late in 1885. "*The first tests in this direction* (*of preventive inoculation*) *were made at the experiment station early in 1886, soon after the hog-cholera bacillus had been discovered.*" Report of Dept. Agr., 1890, p. 110 (issued 1891).

In the report of 1884 that same department said: "In a former report have (been) given details of experiments; which demonstrate beyond question that the microbe of swine plague (H. C.) is a micrococcus, and the evidence furnished was all that could reasonably be required to decide a scientific question of this kind." p. 222.

"In the many cultivations which I have made from material obtained from slaughtered animals, I have never found bacilli except in a very few cases, where the virus was not obtained until after contact with the air, where the vacuum tubes had not been properly sealed, or where the animal was not slaughtered until in the last stages of the disease," p. 225.

Certainly these quotations amply justify the severest criticism of a department of government issuing such reports to the public that created it. If the bacillus of hog cholera (swine plague) was not discovered until late in 1885, certainly the micrococcus of 1880 to 1885 must have been a dream, and the "evidence furnished" anything in value than that which was claimed for it. The most exact and unbiased search over the experiments reported by the government in proof of the fact that the germ of swine plague (H. C), was discovered and demonstrated as such by them, will utterly fail in revealing that fact. They stand to-day in no more favorable position as actual demonstrators of that germ than did Dr. Detmers in 1880. There is not an iota of proof that they discovered the germ even (except their say so) in the report of 1885.

It has been said that my denunciation of the germ of 1885 as a "forgery" is false. A "forged" note, though false in description, may bring the money at the time. Let any one who has carefully investigated this question, and stained the bacilli of this disease, compare the illustration and description given in the Agricultural Report of 1885, p. 212, "*the darker portion is not localizea at the two extremities as in the bacteria of septicæmia in rabbits, but*

*is of uniform width around the entire circumference of the oval,"* with his own observations, and then with the illustration, "Fig. 2" (comparing Fig. 2 with Fig. 1) of Plate (Special Report on "hog cholera," 1889), and say whether that description and illustration in report of 1885 is false or not. Let him remember that in 1885 the government distinctly says, "*the darker portion is not localized at the extremities as in the bacteria of septicæmia in rabbits.*" What, then, can any unbiased and honest man say, who, in 1886, and since then, reads that it is stained at the extremities as in the bacteria of rabbit septicæmia, and is illustrated as so staining?

When does a man receive credit for the discovery of a germ, when he gives a description by which no one can recognize it, or when he gives a correct one with a recognizable illustration?

FIG. 1.

FIG. 2.

The latter is the accepted view, as everyone must admit! Then according to that, instead of discovering the true germ of swine plague late in 1885, the Government did not discover it until 1887, because as is the universal custom, the report of 1886 was not issued until late in the spring of 1887. All I ask of the scientific world is the customary verdict in all such cases. I did not discover the germ of swine plague first; Dr. Ditmers did that. All I ask, in the name of common honesty, is justice to all. Do not forget that even now it cannot be shown from the reports that the Government investigators have exactly and unquestionably demonstrated by scientific experiments that the germ of hog cholera, as they now call the real swine plague, is to the actual cause of that disease.

Next, in order to militate against the value of preventive inoculation, the Agricultural Department announced a new and wide spread swine plague in its report of 1886, but up to to-day has utterly failed in accurately demonstrating that a germ, accidently found in swine (when diseased with the real plague) and other animals, whether diseased or not, even causes disease of any kind, *of itself*, as a natural infection in swine or other animals.

The reader may ask himself, "Why on earth is Billings thrashing all this old straw over again?"

*Merely to show the absolute unworthiness of belief in any assertion of the Agricultural Department regarding a specific germ in causing any disease, unless that assertion is supported by the investigations of men known to be honest and reliable investigators.* The reliability of any statement must always largely depend upon the record of those making it for trustworthiness, unless we absolutely known it to be true from our own observations.

As for the past six years, I have been most bitterly attacked which attack seriously reflects on the honor and intelligence of the people employing and supporting me, and as these attacks have lately had the personal support of the Secretary of Agriculture, I now desire to place before the world all the testimony known to me referring to the acceptance of the Government's position, by those who have given an intelligent consideration to the question.

### THE AUTHORITIES ARE ALL AGAINST THE RELIABILITY OF THE GOVERNMENT'S REPORTS.

In his letter to Senator Paddock (since published and scattered, broadcast by the Department) the Secretary of Agriculture says of Dr. Billings:

"He finally demanded a commission of scientific men to decide whether or not he had shown the investigations of the Bureau to be unreliable, whether the reports of the Bureau were true or false, and whether, to use his own words, he himself was or was not a fraud. On the request of the National Swine Breeders' Association, such a committee of scientific men was appointed, . . . . it was constituted according to Dr. Billings' wishes; it was pronounced by him to be satisfactory, and yet, after a long and careful investigation, it indorsed the investigations of the Bureau of Animal Industry in every essential particular, and in every essential particular decided that Billings was wrong, and intimated that his methods of work were not all that was to be desired Not satisfied to allow the controversy to drop here, he issued a pamphlet entitled 'Evidence Showing that the Report of the Board of Inquiry Concerning Swine Diseases was Fixed,' in which he attacked the honesty and veracity of the honorable scientific gentlemen composing the commission."

This letter to Senator Paddock should be published in full, as if correct, the writer of this article is indeed a fraud, not only as an investigator, but on the people of the United States and the world in general, but it will be deferred to an article on inoculation. This is not a personal quarrel by any means. On my part it is a bitter battle for honesty in public office; a desperate fight against machine politics and its corrupt officials, and so far as I can, I mean to expose every iota of it to public view. The following criticism on the Report of the Board of Inquiry most certainly demonstrates beyond all question, that the Hon. Secretary of Agriculture has been imposed upon, and that the verdict of the world is directly contrary to what he thinks it is.

THE OTHER SIDE OF THE QUESTION.

"The Agricultural Department of the United States named a commission, composed of Shakespeare, Burrill, and Bolton, to settle the question between Salmon and Billings, in connection with the pestiferous diseases of swine in the country, and to make proper investigation, and report on the same. The commission could not complete the task in the time given them, which fact they mention in their report. Nevertheless the government published the report, and accepted its conclusions. The individual experiences of the commission in the diseases in question are small, especially insufficient, and of extremely problematical value are they in relation to the 'swine plague' (Salmon's) or infectious pneumonia. Notwithstanding the assertion of the commissioners that they based their conclusions on personal observations, and notwithstanding certain positive statements in their 'conclusions,' the report gives the impression of uncertainty from want of certainty, and a sufficient scientific foundation. The conclusions appear, therefore, to be statements which have only the worth of insufficiently founded suppositions, and their correctness cannot be substantiated except with difficulty. * * * As to the priority of the discovery of the germ, and the quality of the scientific work, the commission took the side of Salmon as against Billings, but also took care to mention that the government could go on with the work without any outside help. Billings' preventive inoculation finds acknowledgement between the lines."—(*Jahresbericht ueber die Fortschritte in der Lehre-Pathogenen Mikro-organismen*, Baumgarten, 1889—Annual Report on the Progress in the Study of Pathogenic Germs, 1889, p. 178.)

In remarks on some of Billings' publications while in Chicago, the succeeding *Jahresbericht*, 1890, says:

"These and other things form the introduction of a pamphlet (by Billings) against the assertion of the swine plague commission that his methods were not correct. He justifies the methods that he used, the spleen of swine, to gain cultures from, and demonstrates that it was carefully done, so that it appears as if no possible objections could be raised against it. Billings blames, apparently with justice, the commission for treating the subject in a very superficial manner, so far as his laboratory and work were concerned." (P. 185.)

In a very careful and critical review not only of the American swine plague literature, but also a detailed comparative study of the germs of the true American swine plague (not Salmon's) with those of swine plague (European) and other diseases of Europe, Dr. P. Frosch, assistant to Robert Koch in the Laboratory of Hygiene, Berlin, Germany, published his conclusions as follows:

"1. The bacterium of hog cholera (Salmon) and swine plague (Billings) are identical.

"2. The same is the cause of the American swine plague, while the proof of the etiological (causal) connection with this pest, of Salmon's germ of swine plague, that is, for the existence of a second ('widespread epizootic disease') plague of equal extent is not sufficient to warrant such a conclusion."—(*Archiv fuer Hygiene*, vol. 9, p. 279.)

On page 247 of same journal, and in the same article, Frosch says:

"All these circumstances (previously considered in detail), that is, the appearance of cholera and swine plague (Salmon) in the same animal and the same outbreak, and the same time, the presence of still other germs in the organ of the diseased animals, and the fact that in such cases reference is made to chronically diseased hogs, as well as the results of the inoculation experiments, justify the assumption that the swine plague bacterium of Salmon is an accidental presence in chronic cases of hog cholera, for which any idio-pathogenic relation to another pest not sufficient evidence has yet been produced."

As the members of the Swine Plague Commission saw fit to condemn the methods by which they supposed I worked all the time, and did say I worked with at the time, as they brought the best practical results, it may be well to state what an entire outsider has to say on the methods of investigation as published by the

Bureau of Animal Industry in its own reports. On this point Frosch says :

"The most important results must be those obtained in swine by the inoculation of undoubtedly pure cultures of the hog-cholera (S.) bacterium, because in that way only can the specificity of the organism be proven. *But it is directly in this relation that one meets with essential insufficiencies* (*in the government work*). Notwithstanding the numerous experiments made by Salmon with pieces of organs and heart's blood, etc., of diseased swine, still those made with pure cultures of the germ are made prominent by their scarcity and generally negative character. The small number of these positive results would not call forth any objections as to their value, were it not that they did not fulfill all those conditions which are essential in deciding questions of this kind. Of the experiments detailed in reports of 1885 and 1886, *there is not one which can be said to be free from objections.* The cause of the same lies, first, in the fact that the order of the experiments was not correctly arranged, the control animals dying first in two cases and at the same time in a third ; while in the feeding experiments of the same year control animals are not mentioned. A second very essential objection to the method pursued by Salmon is the manner he went to work to obtain pure cultures, * * * *the consequences of such an unscientific method are to be distinctly seen in the report itself*, when, mixed with the inoculated germs other 'large' or 'fine' bacilli are mentioned as being present. The best proof of the unscientific character of his method is given by Salmon himself in the assertion that cholera and swine plague can occur in the same animal, a connection which absolutely demands in each case the use of the plate method" (*Ibid*, p. 243.)

To this detailed study and exact report of Dr. Frosch's the government investigators most seriously objected, as would be natural, and one of them made reply thereto in the same Archiv (*Zeitschrift fuer Hygiene*, vol. 10.) To the same Frosch answered as follows :

"The foregoing article by Dr. Theobald Smith, and especially his comments on a contribution of my own, induces me to once again place my position regarding the American swine plague clearly before the world. To begin at once with the point that seems to have mostly irritated Smith, I do not think that any one but he can find in my former article any special partiality or unjust discrimination in favor of the investigations of F. S. Billings Such an estimate of my contribution to the question is only possible to a person who has cursorily read the same, or who is profoundly ignorant of the character of the hygienic institute at Berlin, and the work which is done therein.

"As I declared in the beginning of my previous article, the reception of the cultures from Billings at this institute, and the request of Prof. Koch, led me to enter upon the study of the American swine plague. The task which I had to undertake was not, as Smith appears to believe, to decide as to whom belonged the most or earliest credit for work done, but to see how far, from a purely scientific point of view, the solution of the real question of the etiology of this disease had been advanced, which, at the time, seemed to be buried in darkness, in consequence of contradictory publications.

"That I should depend more upon my own investigations with the cultures at my disposal than upon the investigations of Billings should not be questioned by Smith. That I should refer to the investigations of Billings, after assuring myself of the identity of his germ and Salmon's hog cholera germ, in order to decide as to the pathogenity (disease-producing power) of the germs in swine, was forced by Smith himself, *for, much as I regret to have to repeat it, the methods and experiments published in the reports of the Bureau of Animal industry (1885 to 1887-8) do not correspond to the scientific conditions necessary to the establishment of a new infectious disease in a manner to be desired.*

"It is by no means necessary for me to repeat what I said in my previous publication, as Smith admits it, in regard to the report of 1885, and, on the other hand, the superficial investigations described in the other reports display so little exclusion and exact employment of Koch's methods to correspond with the importance of the assertion of the appearance of a new exciter of infection (germ), closely related to the swine plague.

"I might here call attention to the fact that even in the work of Smith, described in his article in this issue of the *Zeitschrift*, the method for differentiating the two germs, the employment of the hanging drop, is not sufficiently reliable.

"Smith also seems to complain that I have considered the mentioned reports of the Bureau of Animal Industry too closely after the standpoint of to-day. Even though we may have to-day new ideas of what a pure culture should be, or substantially other methods of obtaining the same than formerly, still it was perfectly justifiable to prove the case as to how the earlier results of the Bureau correspond with these newer ideas.

"As shown in my previous publication, it is evident from the report of the bureau that at that time Koch's methods were well known there, and I do not think that it is demanding too much of the bacteriologists of the bureau to assume that they are acquainted with the methods as published in their reports.

"As to the slur upon Billings' work in Smith's publication, I can safely leave it to the former investigator to consider them. I have only to refer here to his inoculation experiments in swine, to which I am inclined to give full credit as reliable evidence, because Billings emphasizes the control of the pure cultures used in the same by other cultivating tests.

"The number of these experiments was sufficiently great to demonstrate the infectiousness of the germs and their specific characteristics. Smith neglects to observe that the experiments quoted by Billings were specially selected out of a great number. I find it remarkable that Smith should question these twelve published experiments of Billings, the value of which is beyond doubt.

"The inference (by Smith) that I allowed Billings' publication to influence me in the consideration of Salmon's swine plague, by no means corresponds with the facts. *For judging this question I have referred to the publications of the bureau on the assumption that in these ffiocial reports the most reliable material must be found.*

"In regard to the question as to whether the Salmon swine plague germ is an independent cause of a specific disease I cannot change my previously expressed opinion. How far I am right can best be judged by reading Smith's publication in this journal.

"As will be remembered, Salmon, supporting himself on the German schweine seuche, distinctly and emphatically asserts the existence of an independent plague, and at the same time united with the hog cholera, which had the same degree of extension over the country.

"Judged by the investigations published in the reports of the bureau, we find but proportionately few cases, and these not free from objections, of the appearance of a disease-producing germ in chronic cases of hog cholera. The conditions closely resemble those seen in certain infectious diseases of man, where the secondary appearance of pathogenic germs has long been observed, without any one asserting them to be independent causal moments, or the cause of extensive epidemics.

"The last two cases reported by Smith in favor of his swine plague do not give evidence which sufficiently excludes the concomital existence of hog cholera also. At the same time and the same place, several swine are reported to have died from the cholera.

"The fact stands for me confirmed, that in the five years that have passed since the second plague was first announced, not one single case of an independent appearance of Salmon's swine plague has been reported, which can be said to be free from objections.

"The mistaken conception of Salmon's place in the swine plague investigations must be laid to the fact that the publications, both on swine plague and hog cholera, have his name, and as such have been quoted in the literature.

"From the present publication of Smith's, however, which could not be seen in reading the reports of the Bureau of Animal Industry, it is evident that Salmon was not the discoverer of either the hog cholera germ or that of the swine plague, so now we know the true condition of things in that regard."

An American reviewer of the entire question, so far it pertains to the publications of the Agricultural Department and the report of the Board of Inquiry, says:

"We have compared the extracts cited by Dr. Billings from the reports of the Department of Agriculture, line by line and word for word ; we have carefully examined the plates accompanying these reports to which he refers, and we have also read the entire context from which he quotes, in order to avoid any possible bias, which may follow from reading disconnected sentences. As a result we are enabled to say that all of Dr. Billings' charges, sweeping and severe as they are, are true and just, and we cannot understand how the committee appointed to investigate the special question involved could fail to make the same examination, to arrive at the same result, and to publish that result in calm, judicious language, not as a matter of justice to Dr. Billings, or of criticism of Dr. Salmon, but as a positive duty to science. Regarded from this point of view, the report of that committee is the most disappointing document of the kind we have ever seen. This we may say, without intimating, as Dr. Billings does, that its authors were under the control of the 'Bureaucratic Whip.'

"The charges made by Dr. Billings, expressed in more conventional language than he employs himself, are that Dr. Salmon's bacteriological work is not expert, that he described a germ of swine plague when he had no such germ in his possession, that he has printed assertions without scientific evidence to sustain them. He even states directly that one micro-organism (the description given by Dr. Salmon) was evolved from the inner consciousness of that gentleman, and designates this procedure by a term to be found in the criminal code, and not usually employed in scientific discussion, however applicable it might be regarded in one sense to evidence, which Dr. Billings states to have been deliberately made up for the occasion.

"Regarding the bacteriological questions involved, we would be inclined to suspend judgment, were it not for two sets of facts: First, the evasions of the 'Board of Inquiry;' *second, the gross logical errors and inherent contradictions of Salmon's writings; the latter are glaring*, it would not have hurt Dr. Salmon's reputation, had he on being convinced of errors in his methods or conclusions, candidly acknowledged them, and accepted the corrections furnished by others and verified by himself. Men of a real rank in the scientific hierarchy, like Virchow, Klein, Koch, and others—and Dr. Billings here sets his opponent an example worth imitating—(No. III, pp. 50–53)—have not hesitated to acknowledge mistakes, such as the most honest and able investigators are liable to in experimental science, as well as in interpreting or quoting the results of others. But Dr. Salmon has preferred to gloss over the matter, and in order to sustain a claim to priority at the expense of another, and to conceal the fact that he adopted the corrections of others, has involved himself in a labyrinth of contradictions; the inevitable consequence of misrepresentation, be they intentional or unintentional. Which of the two classes Dr. Salmon's belongs to, no one who reads Dr. Billings' pamphlet and verifies its citations can for a moment doubt."—(*Journal Comp. Med.*, vol. X, p. 399.)

Again the same reviewer says:

"Regarding the bacteriological side of the question, the commission is equally unfortunate, and although the majority report claims that on the substantial point a dissenting minority report by Professor Bolton is in accord with their own, we do not think our readers will agree with them. Professor Bolton says:

"'*During my work as a commissioner I have failed to meet with an epizootic which I am satisfied was what is termed 'swine plague' in the Bureau reports, though previous to my appointment on the Board I studied one such outbreak.* In this case, however, I directed my attention to the bacteriological questions exclusively, and I am therefore unable to pronounce on the difference in the pathological lesions in the two diseases. But I am not inclined to attach any great importance to these differences as set forth in the reports. The description otherwise I find correct and well stated. In my investigations as commissioner I have been able to find but one organism which in my opinion caused the outbreaks under examination, and that I regard as identical with the hog cholera germ described in the reports of the Bureau, and I find the description therein given correct. As will be inferred from what has gone before, I feel sure that another organism, correctly described in the reports as the "swine plague germ," is found under circumstances which render it highly probable, if not certain, that it also causes disease. As to whether these two organisms are always present and operate together to cause disease, or whether the two are merely varieties of the same germ, must be decided by future investigation.'

"The main report insists that there are at least two widespread, distinct diseases which are caused by distinct micro-organisms. If this is what the committee calls agreeing, we would like to know what disagreement is. Who will be in doubt for a moment as to who, or what induced Professor Bolton to write the last few lines of his report. They are merely loop-holes for the escape of the man through whose hands the report went. Whether pity or courtesy were Professor Bolton's motives, he owed a higher duty to the country than to Dr. Salmon; we regret that he was not less ambiguous in expressing his convictions, although as contrasted with the majority report we have occasion to be grateful for even this much."

"FOOLING THE SWINE BREEDERS."

There is a well-developed and continually growing belief among the great body of American swine breeders that they have been badly fooled recently—in short, they are commencing to look upon that meagre report of the so-called special commission on diseases of swine as a fiasco of the first water. 'Twas with not a little satisfaction that they regarded the appointment of the said commission. Were high-toned scientists to be relied upon the material of the Board was surely satisfactory, and an appropriation of $30,000 for their expenses seemed ample to produce tangible results.

When the august body got into line of work December, 1888, swine breeders the country over were perfectly willing to wait until April 1 for the momentous findings expected. When April fool's day came they were, according to the custom of the day, fooled. The report came not. Premonitory rumblings of the approaching verdict were now and again heralded through the press. The mountain of scientific knowledge—the Parnassus of bacteriological learning—travailed in labor and brought forth—a mouse ! The insignificance of the commission's production, so far as its intrinsic value to a long-suffering fraternity of breeders was concerned, was certainly in the proportion indicated, and any iota of value it may have contained was destroyed by the deceptive method of its publication.

A "boiling down" editorial bureau had been established at Washington and the commission's report was probably the initial work it edited. The press of the country looked for assistance from the said editorial bureau, and prepared itself implicitly to receive and publish as official and authentic the edited advance proofs the said Bureau was paid to disseminate. Thus when about the second week of August a publication purporting to be the true and correct finding of the swine commission arrived from Washington, it was immediately published in full and commented upon as the *in extenso* report. The *Farmer's Review* was not fooled, but in a short editorial outlined the weaknesss of the report, and since then has had no occasion to change its assertions. They will, however, bear considerable elaboration.

The Department of Agriculture editor, or mayhap some one more closely interested in the verdict of the commission, sent out the preliminary portion of the report with an explanatory introduction in which occurred the following statement :

"We subjoin here the conclusions attached to the report of the commission, and signed, owing to the absence of Prof. Bolton, by Dr. Shakespeare and Prof. Burrill. Prof. Bolton, however, *furnishes a supplementary report practically confirming the report of his two colleagues.*"

The italics are our own, used to emphasize the assertions that Prof. Bolton's report confirmed that of the other commissioners. We shall see whether that was the case ; but first let us ask, why, believing this, Prof. Bolton's report was not sent out at the same time with the conclusions signed by Professors Shakespeare and Burrill ? That it was not, leaves the swine breeder to accept the unpleasant inference that it was held back to give the conclusions the most favorable character as concerning Dr. Salmon and his Bureau of Animal Industry.

This subterfuge has, however, proved unavailing. It has been recoiled in hurtful strength upon those who employed it. The ire of the breeders and of the bulldozed press has been aroused since receiving the official report in full, of which Prof. Bolton's contribution is perhaps the most important portion. The result must and certainly will be an unanimous demand for clean work in high places, for just value for public money spent, and for actual, honest, practical research in behalf of the inestimably important swine industry of this country.

As mentioned in a previous editorial in these columns, the commission's conclusions, as sent out by either the editorial bureau at Washington, Dr. Salmon, or the Bureau of Animal Industry, declare that there are two prevalent diseases of swine in this country, *i. e.*, "hog cholera" and Dr. Salmon's "swine plague." The former disease has in all conscience been bad enough. Our swine have had a sufficiently damaging character given them as regards disease. The industry has been hurt thereby. Germany, for instance, has barred out our pork, presumably on account of disease. The patriotic authorities at Washington surely forgot these things when they, with all their power of official advertising, solemnly declare that another serious disease is rife among our hogs. The breeders will thank them for this, doubtless. They will regard their $30,000 well spent in accomplishing such results. They will appreciate government aid in fostering their interests. They will admire the self-conceited confidence of a commission which, nothing daunted, proclaims to the wide world a new disease among our hogs without offering one solitary fact to substantiate the baneful assertion. Time will show what they really think.

And Prof. Bolton confirms this advertising of "swine plague," does he? Let us see. A comparison of the "conclusions" of Profs. Shakespeare and Burrill, with the independent minority report of Prof. Bolton, *in re* "swine plague," will enable the swine breeder to arrive at a correct conception of the truth of the matter. (See next page.)

If these to reports corroborate each other we fail to see it, and we feel sure that breeders find themselves in the same position.

In addition to getting up this wholly unwarranted "swine plague" scare, the commission seeks to throw cold water on legitimate and praiseworthy attempts to prevent "hog cholera," by inoculation with carefully prepared virus of the disease. Totally unable to destroy pigs thus inoculated, either by exposing them to an outbreak of cholera, or by feeding them with germ cultures, it warns breeders against inoculation as a preventive, on the grounds that it may tend to spread the disease. The points made against inoculation are those long recognized and time and again written about by Dr. Billings, who considers them of little moment if proper precautions are taken in carrying out inoculation.

### CONCLUSIONS.

It is the opinion of the commission, based upon their own individual observations and examinations of the subject, that *there are at least two widespread epidemic diseases of hogs in this country* which are caused by *different micro-organisms*, but which have a clinical history and pathological lesions more or less similar and very difficult to distinguish without the aid of the microscope, and resort to bacteriological methods. * * * So far as the knowledge and the observation of the commission go, one of the epidemic diseases, viz., that called by the Bureau Authorities, 'swine plague," appears to be far less prevalent than the other which has been named by them "hog cholera." The commission are further of the opinion that the disease called by the authorities at Washington "hog cholera" is caused by the specific action of a certain microbe named by them "the hog cholera germ," which has certain characteristics of form, size, movement, mode of growth in artificial cultures, and action upon certain lower animals, and taken together enable one to distinguish it from the other microbes which have been described from time to time by various authorities as present in swine disease ; and that the descriptions of this microbe and its peculiarities, as set forth in recent annual reports of the Bureau of Animal Industry, are fairly accurate. The commission are of the opinion, *although to a less positive degree*, that the epidemic disease called by the Bureau authorities "swine plague," has as its specific cause a certain microbe possessing characteristics which have been fairly well described in recent annual reports of the Bureau of Animal Industry, which distinguish it both biologically and pathologically from the first mentioned "germ of hog cholera."

### PROF. BOLTON'S REPORT.

During my work as commissioner *I have failed to meet with an epizootic which I am satisfied was what is termed "swine plague"* in the Bureau reports, though previous to my appointment on the Board I studied one such outbreak. In this case, however, I directed my attention to the bacteriological questions exclusively, and I am therefore unable to pronounce on the difference in the pathological lesions in the two diseases. But *I am not inclined to attach any great importance to these differences* as set forth in the reports. The descriptions otherwise I find correct and well stated. In my investigations as commissioner *I have been able to find but one organism* which in my opinion caused the outbreaks under examination and that I regard as identical with the hog cholera germ described in the reports of the Bureau, but I find the description therein given correct. As will be inferred from what has gone before, I feel sure that another organism, correctly described in the reports as the "swine plague germ," is found under circumstances which render it highly probable, if not certain, that it also causes disease. As to whether these two organisms are always present and operate together to cause disease, or whether the two are merely varieties of the same germ, must be decided by future investigation.

The fact that the inoculated pigs from Nebraska proved immune against hog cholera, as demonstrated by the commission, is practically the only valuable thing given to the public in return for the $30,000 expended.

The swine-breeding fraternity cares little or nothing about the *pros* and *cons* of learned squabbles about germs. It is interested in finding some method of preventing or alleviating the heavy annual loss from swine disease. Naturally enough it has looked to the Bureau of Animal Industry for help in this direction, but for the thousands upon thousands of dollars spent presumably in their interests they have reaped not a single *original* fact of real practical value in their everyday business. Time, indeed, that the work of such a bureau were thoroughly inquired into! Or, better still, that the government should recognize the fact that little but unintelligible bulletin publishing has hitherto been accomplished, and that the time has arrived when practical work is imperative.

Let the swine breeders of America unanimously demand such work. Let them hang no longer upon the words of false prophets or consent to be blindly led by the blind. Write off as lost the vast sums of money already expended in the research of swine diseases, and commence anew, and that now. The bureau has demonstrated its inability under its present head to serve the swine-breeding industry. Commissions are evidently a delusion and a snare. Other means and methods must be employed, and it is for the swine breeders of the country to demand the change in no uncertain voice.

Let the best men to be found the world over be employed forthwith, if possible, not as special commissioners, but each in his individual capacity, to investigate swine diseases in the different parts of this country. Let each man work for his own honor at the expense of the government, and offer them inducements for the discovery of disease cures or preventives. In this way good results will speedily be arrived at in place of voluminous and valueless bulletins offered to the long-suffering public in place of tangible duties performed at the public expense.

The *Farmers' Review* has come to the conclusion that swine breeders must put a stop to all this fooling they have been subjected to and demand and obtain the Government aid in regard to swine diseases, which is their right. This matter is one which demands united, energetic, enthusiastic agitation. Let the breeders strike while the iron is hot.—*Farmers' Review*, *Chicago*.

"A QUESTION FOR THE COMMISSION."

"The Report of the United States Board of Inquiry Concerning Epizootic Diseases Among Swine"—the conclusions of which were submitted last week—has been received.

The letter of instruction to these gentlemen, signed by Commissioner Colman, was unmistakably drawn to direct the investigation of the scientists to the points at issue between the Department of Agriculture on the one hand in the persons of Dr. Salmon and his assistants, and the Universities of Nebraska and Ohio on the other as represented by Dr. Billings and Dr. Detmers. The commission was specifically charged to determine whether the work of the Department was accurate and original, both of which points were in controversy raised by Drs. Billings and Detmers. That done, independent investigations were to be undertaken. The disputes between the doctors named, who have been engaged in this line of investigation, involved several points in bacteriological research—with which the *Gazette* and other laymen have nothing to do—and a vastly more important issue—namely, whether there are one or two distinct swine plagues. The Department affirmed there are two—the one "hog cholera," with its specific germ and the seat of the disease in the intestines; the other, which is named "swine plague," with a distinct germ, the resultant disease of which finds lodgment in the lungs. The other investigators denied the existence of more than one disease and one germ, claiming that the work of this pest germ was at times manifest in the intestines, at others in the lungs, according to certain conditions.

Such was the chief subject of dispute. As stated in our last issue, on all points raised the commission has determined in the Department's favor. *And yet there is an indefiniteness about its report that is very unsatisfactory.* It declares that there are two widespread epidemic diseases of hogs, and that their descriptions by the Bureau are fairly accurate, "except it does not appear that 'hog cholera' of these reports can be said to have its special and exclusive seat in the digestive tract of the animal as distinct from the lungs." In view of all the circumstances this appears to be a very significant exception. "Swine plague"—the "new" disease—is pronounced far less prevalent than "hog cholera." After confirming the Department's conclusions as to "hog cholera" and its germ, the commission turns its attention to the other diseases thus:

"The commission are also of the opinion, *although to a less positive degree*, that the epidemic disease called by the Bureau authorities 'swine plague' has as its specific cause a certain microbe possessing characteristics which have been fairly well described in recent annual reports of the Bureau of Animal Industry, which distinguish it both biologically and pathologically from the first mentioned 'germ of hog cholera.'"

Is this the language in which science affirms its conclusions—"to a less positive degree"? Again, Dr. Bolton, the third member of the commission—who files a supplementary report—submits the following:

"During my work as commissioner I have failed to meet with an epizootic which I am satisfied was what is termed 'swine plague' in the Bureau reports, though previous to my appointment on the Board I studied one such outbreak. In this case, however, I directed my attention to the bacteriological questions exclusively, and I am therefore unable to pronounce on the difference in the pathological lesions in the two diseases. But I am not inclined to attach any great importance to these differences as set forth in the reports. The descriptions otherwise I find correct and well stated. In my investigations as commissioner I have been able to find but one organism which, in my opininion, caused the outbreaks under examination, and that I regard as identical with the hog cholera germ described in the reports of the Bureau, and I find the description therein given correct. As will be inferred from what has gone before, I feel sure that another organism, correctly described in the reports as the 'swine plague germ,' is found under circumstances which render it highly probable, if not certain, that it also causes disease. As to whether these two organisms are always present and operate together to cause disease, or whether the two are merely varieties of the same germ, must be decided by future investigation. The differences between them, as pointed out by the Bureau, are sufficient to compel us to treat them as different germs, however perplexing it may seem that two micro-organisms are capable of producing such similar or, it may be, identical lesions."

Be it observed that although Dr. Bolton has not in all his investigation the country over as commissioner discovered one single case of this second disease—"swine plague," he yet "feels sure" that another germ is found under certain circumstances which render it "*highly probable*, if not certain," that it causes disease! On what evidence is he "sure" that it is "highly probable"? Is this again the language with which scientists seek to saddle a *second* swine plague on the country? The *Gazette* cannot be charged with hypercriticism of scientists, but it confesses it is unable to appreciate the force of Dr. Bolton's statement of the situation.

In fact, the definiteness of the commission—"to a less positive degree"—as to the existence of a second swine plague, should have led it, the *Gazette* believes, to submit conclusive evidence to the country on this point. That a germ has been found which the department believes is the cause of its "swine plague," does not admit of doubt; the question arises, however, is this the germ of merely a local, non-infectious, non-contagious disease, or is it a genuine *pest?* If the commission has answered this query conclusively, the *Gazette* has failed to observe it. "Hog cholera" is admittedly a plague; its germs, fresh from the blood or cultivated in artificial media, infallibly produce the disease when introduced into the systems of healthy swine. If "swine plague" is a pest, its germ should likewise produce that disease with its specific lesions —but as we read the commission's conclusions, it can scarcely be said to have specific lesions. Now the commission had in its charge since February last, nearly twenty hogs, which, in Nebraska, had been inoculated against "hog cholera," and they withstood every attempt made by the commission to kill them by exposure to virulent natural outbreaks, or by inoculation with excessive doses of the germ of "hog cholera." They were "hog cholera" proof, as the commission admits. But *these hogs were not tested with "swine plague" germs or exposure.* And the question which the *Gazette* respectfully asks the commission is, Why not? Here was the opportunity of all to demonstrate beyond all cavil the existence of a second *pest*, for if these hogs had succumbed to inoculations of "swine plague" germs. Dr. Billings would have been proved conclusively a charlatan, an ignoramus, and a frand on his own chosen ground. He defied the commission to kill his inoculated hogs with any contagious or infectious swine disease; it could not kill them with "hog cholera;" if a second pest—"swine plague" exists, why was not its existence proved thus undeniably? If it kills swine as a *pest* these hogs should have succumbed to it, as they had not been given immunity from its germs, and the commission was in possession of the material with which to infect and kill them if it could. Why was this not done?

The *Gazette* asks this question in all sincerity. It has the utmost respect for the scientific attainments of the members of this commission, and it cares not one picayune whether this or that investigator goes down in the final determination of this much-discussed question; it is interested solely in learning the *truth;* but when it is asserted that two swine pests exist, the matter of the prevention of the disease by inoculation becomes necessarily so complicated as to be almost, if not quite impossible of attainment, and before that conclusion is definitely reached, swine-breeders have a right to demand unconditioned proof. Why was it not offered when the opportunity presented?—(*Breeders' Gazette, August 21, 1892.*)

### HOG CHOLERA.

The following investigations were begun in November of last year, and have been continued up to the present time. We were prompted to undertake them, not only on account of the scientific interest involved, but also on account of the news of many disastrous outbreaks of the disease in various parts of the State. In the course of our work we have visited many widely separated portions of the State, and encountered the disease in all its stages. In some herds the attack had just commenced; in others, we saw only cases of long standing, and, as will be seen elsewhere, the disease presents very different features in the two stages. We also found it in mild as well as in virulent form. In fact, in some cases very few animals died, whereas, in others, there were no recoveries. The majority of the outbreaks were of a very severe type. So we have been able to observe the disease in all its aspects.

We visited various portions of the State and took along all the best apparatus and appliances for our work. Material collected in this way was brought back to the laboratory and thoroughly examined. The germs which we found were cultivated and grown on suitable materials, and were tested upon mice, rabbits and hogs. We obtained them from the spleen, liver, blood, and intestinal ulcers. In some cases we failed to get any germs at all, and although we obtained in other cases several different kinds, we have no reason to believe that more than one of them is concerned in the disease.

The only other organism deserving special attention is one which we regard as identical with the bacillus of hog cholera of Salmon, in the report quoted above, which the reader is referred to for a complete description of the same.

We have not been able to produce the disease by subcutaneous inoculations of even quite large amounts (5 c. c.) of bouillon cultures of this bacillus, but we have succeeded by feeding 300–500 c. c. bouillon cultures in milk made alkaline with carbonate of soda.—(Bulletin No. 6, New Series II, July, 1888, South Carolina Experiment Stations.)

At the meeting of the United States Veterinary Medical Association, held at Washington, September, 1891, Dr. A. W. Clement made some remarks which bear on the report of that "Board of Inquiry," because "It has been my (C.'s) opportunity for the last three or four years of doing some work in that line myself, in association with Professor Welch, of Johns Hopkins University. I might say, in parenthesis, that my work in this line has nothing to do with my position as Government Inspector. As to what connection the organism (Salmon's swine plague germ) has with the lesions described in the reports of the Bureau, is a question on which we all might not agree. Nevertheless the swine plague organism does cause trouble. The trouble in hogs is, as a rule, in our experience, one of mixed infection. We have not had the opportunity of seeing an outbreak of swine plague (Salmon's) pure and simple. We have found that it is very hard to say when swine plague (Salmon's) is present that hog cholera (Salmon's) is absent, from the fact that swine plauge kills (What, rabbits or hogs? B.) in a few hours, while hog cholera requires some days. If, then, an animal be killed and presents lesions in the intestines, as are generally supposed to be characteristic of hog cholera, the statement must be very carefully considered before it is made, that hog cholera is not present. We were thrown off our track during the earlier part of our investigations. We found afterwards that hog cholera did exist in these animals that we thought had swine plague (Salmon's) pure and simple. I would simply say in a general way that from our investigations we have found Dr. Billings is right in certain other matters."—(*Journal of Comparative Medicine* vol. XII, p. 549.)

In order to show how low these Department investigators have fallen in political degradation, and how they have begged for the patronage and support of the leading editors of the Agricultural Press of this country, I hereby append one of several letters of the same nature, which have been sent me, and with it the answer which the writer received. It may open the eyes of my European confreres to "ways that are strange and things peculiar" in that Heathen Chinee, the political investigator, in the United States.

U. S. DEPARTMENT OF AGRICULTURE, BUREAU OF ANIMAL INDUSTRY.

WASHINGTON, D. C., April 9, 1890.

Henry Wallace, Esq., Editor of the *Homestead*, Des Moines, Iowa, Dear Sir:—For some weeks I have been receiving a copy of the *Homestead* marked "complimentary" for which I wish to return thanks, presuming that it comes from you. I have not been a reader of your paper before because it did not come to my desk, and, on account of the many duties devolving upon me, I seldom get time to consult the periodicals on file in our library. Mr. Hill, who has charge of the editorial division of the department, has frequently spoken to me about you, generally in connection with the attitude which your paper has assumed toward the Bureau of Animal Industry, when discussing the subject of inoculation as a means of preventing hog cholera, and he has always referred to you in such complimentary terms that I venture to address you this personal letter in the hope that any misunderstanding which you may have of our position may be explained, and that we may, if possible, obtain your good will even though our opinions may continue to differ.

The Bureau of Animal Industry is endeavoring to work for the best interest of the stock owners of the United States, and we are earnestly striving to furnish information which shall be as nearly as possible impartial and reliable. It take it for granted that the *Homestead* is trying to treat its readers in the same manner. We therefore meet on common ground; we are working in the same field, and if we differ in our opinions on certain questions, is that any reason why one should publicly refer to the other in such disrespectful terms as to tend to destroy his influence and usefulness in the work in which we are both interested? Is it possible that the farmers' cause can be promoted by those who are in the field wasting their energies and in destroying one another? I ask these questions frankly because I believe you are a reasonable man, broad enough and liberal enough not to be offended by them. Bear with me, if you please, while I make a few remarks inspired by the short article on page three of the *Homestead* of April 4. That I was not prejudiced against inoculation is shown by the fact that I was the first in the country to test it, and that before Dr. Billings began his investigations in Nebraska I had worked out the method of cultivating the virus, the proper dose to use for inoculation, and the effect following this operation. And in the report of this Bureau for 1886, pages 60 to 70, the details of the experiments are given so fully that any one who can make a culture of germs can repeat the experiment for himself. Against my own hopes I was compelled to decide that inoculation was not a satisfactory method of preventing hog cholera. No one could be more anxious than I was to offer some solution of the hog cholera problem to our farmers, and if I yielded my opinion to the inexorable logic of the facts, should I be censured for it? Admit, if you please, that I was wrong in my conclusion, would even that be sufficient to justify the language which some of the agricultural journals delight to hurl at me and at this Bureau on account of the stand which I have taken?

The experiments which I have made have been planned to bring out the truth and the details have been published. That they show inoculation cannot be depended upon to protect from hog cholera under the conditions of these experiments, is plain. It is possible, however, as I have freely admitted, though hardly probable, that the conditions of exposure on farms are not so severe. In that case a degree of protection which in our tests was insufficient might ward off the disease in most cases on farms. But surely, this ought to be demonstrated before farmers are advised to risk their animals and spend their money in having this operation performed, and this is what has been done in any case by Dr. Billings. He has withheld the details of his tests, he has concealed his failures and he has made claims which the facts do not justify. Take his experiment in Nebraska, where had 1,000 hogs inoculated. Though he admitted at the time that about 400 of them afterwards died of hog cholera, he now says in his pamphlet that there was a "a reported loss of only eleven out of the whole number." Can you recommend inoculation from that experiment, in which 40 per cent. of the hogs died of hog cholera? If not, where is the evidence which favors it?

Take the experiment in the article in the *Homestead*, to which I have already referred, where you say "This is the kind of proof required." Certainly you could not have scanned the details of that experiment very closely, because there was none of the conditions observed which would give reliable results. Consider simply the two inoculated hogs. I have it on good authority that they were the survivors of a lot of hogs which had been affected with cholera. Is it not presumable that in such a case, having resisted one outbreak, they might be expected to resist another without inoculation? In other words, when a herd of swine is affected the most susceptible animals die and those which live have more than an average power of resistance.

The proper way to make an experiment is to take a lot of twenty-five or fifty hogs which have not been exposed to the disease, inoculate half of them, then expose all in exactly the same manner to the contagion; keep all under the same conditions and note how much better the inoculated lot withstands the disease than the others. By making the kind of experiments which Dr. Billings reports one can prove anything, especially if he conceals his failures.

I have written much more than I intended to, but I trust you will not be bored by it. The subject is an important one, and I feel sure that neither the *Homestead* nor any other paper can afford to either intentionally or unintentionally mislead its readers in regard to it. In my report on inoculation, of which a copy is mailed you, I have endeavored to place before the people the unvarnished facts according to the evidence now at hand, and before the conclusions therein expressed can be changed, there must be additional experiments made which are properly conducted, and which yield different results. I should be pleased to see some of the experiment stations take the matter up, and would assist them in so doing in any way in my power.

With the hope that this letter will not reach you on your "busy day," I am, very respectfully,

D. E. SALMON.

DES MOINES, IA., May 6, '90.—Professor D. E. Salmon, Bureau of Animal Industry, Washington, D. C , Dear Sir—Your favor of April 9 came to the office while I was absent on a five-weeks' business trip, and I take the first moment of leisure after my return to reply.

Allow me to thank you for the spirit and tone in which your letter is written, and to express the conviction that there is no need for men who differ honestly on certain measures of public policy to treat each other any other way than as gentlemen.

I may as well say to you frankly that the practical results of the investigations of the Bureau of Animal Industry with reference to hog cholera have not warranted any great hopes on the part of the swine growers of America. Possibly it is no fault of the bureau. Nevertheless, it accounts for the attitude which many of the farmers of the West sustain toward it and the favor with which they are inclined to receive the views and promises of Dr. Billings. It must be conceded that Dr. Billings has to a great extent the confidence of the leading agriculturists of the West, and what is more remarkable, the confidence of the leading stockmen of Nebraska. I attended their meeting in Lincoln in February, and was more than surprised when they elected him, though not a resident of the State, President of the Nebraska Stock Breeders' Association for the coming year.

As I understand it, whether Billings' theory and practice of inoculation is correct or not must be determined by the facts. When the bureau of animal industry took the position that there was no remedy for the hog cholera, and the government failed to take measures for stamping out the disease as they did the pleuro-pnemonia in cattle, it is not surprising that the farmers should turn for relief to Dr. Billings who gave assurance that under certain conditions he could prevent the disease, and proposed to let the practical results of his work determine the correctness of his theory. The bitter warfare that has been made on Dr. Billings and which is believed by his friends to come largely from the Bureau of Animal Industry, only intensifies the popular feeling on his behalf, and it would seem to me that if the Bureau of Animal Industry through its agents or employés has been at the bottom of, or accessory to, this warfare, the best thing for all concerned is that it should cease. If Billings is either a fraud or an unbalanced enthusiast, time will very soon tell the story. If he is not, but has discovered a method by which under certain circumstances and conditions, hog cholera can be prevented, then he is entitled to the credit.

Some of the statements you make differ from my understanding of the facts. It is conceded, I believe, by Dr. Billings' friends that 400 hogs inoculated were actually diseased, died of cholera, but it is not conceded that these 400 are part of the thousand to which you refer. I might mention other discrepancies, but have not leisure at this time.

I confess to you that the report of the special commission did more to prejudice me against the Bureau of Animal Industry than any other one thing. You will pardon me if I say that it seemed to be a whitewashiug affair, sedulously, to all appearances as seen by an outsider, concealing facts and failing to make the investigations which it was appointed to make.

I have not, since my return, had time to go into the controversy between you and Dr. Billings fully. I stand ready to give the Bureau of Animal Industry credit for all the good work it actually performs and to insist, as I shall do in one of the coming issues of the *Homestead*, on the enlargement of its powers with reference to the dealing with pleuro-pneumonia in New York. I also stand ready to give Dr. Billings credit for any results which he may accomplish, the object in both cases being to secure as far as possible the financial well being of the constituents of the *Homestead.*

Very truly,

HENRY WALLACE,

*Editor Homestead.*

Now, I honestly ask what is the value of scientific assertions of, so called, investigators of the character represented by the foregoing quotations? I ask this because, as will be at once shown, assertions of the same nature have been made in the Reports of the Department of Agriculture, and elsewhere, by the same individuals, in relation to our discovery of the bacillus *Southern Cattle Plague.*

They absolutely deny its existence. In order that there may be no misunderstanding in the matter, the literature, in so far as it pertains to the specific cause of the disease will be placed before the reader. As has thus far been the case in connection with all the germ discoveries of the investigators of the Agricultural Department, the inciting movement leading to the so called discovery has invariably been borrowed from European investigators.

1st. We have the "micrococcus" of Pasteur's "Rouget" as the cause of our swine plague from 1880 to 1885.

2d Schülz work on a swine disease in Europe leads to the false description of the germ of swine plague in 1885 (Report of) and then the fortunate discovery of an organism closely resembling that of Schülz misnamed "Schweine-Seuche," by the Department investigators, leads to the assertion of a second "widespread epidemic disease among the hogs of this country,"—a second swine plague which, as such, does not exist.

3d. Almost identically the same thing has happened in connection with the Southern cattle plague and its etiology. The work of Laneran and others had shown that certain *Protozoa* were in some way related to fever and ague and similar organisms, as indicated by Koch, had been found in the blood of birds and other animals living in, especially malarial districts. It is true that their etiological relation to any specific disease is a mere hypothesis, but the discovery came in very handy, and as some kind of an object, which colors and that is all there is to it, but which is in all probability some sort of coagulated necrosis of the plasma of the red-blood cells happened to be found in the blood of Southern cattle. To see such a thing was all that was necessary. It was there in some condition or another. It mattered not whether it could be cultivated, whether it could be isolated and inoculated. Something was there and it was all that was necessary to use as a political militant to offset and disqualify the Nebraska investigators. Foreigners cannot see that unless the Agricultural Department at Washington can face the people and Congress and make them think that the investigators are doing all the work and the only correct work, that Congress will not support that institution, *because its power as a spoke in the political machine would be void.* That is the meat in the cocoanut in this dispute, so far as that department is concerned.

The first publication of this kind emanating from the Agricultural Department, was an article in the "Medical News"—Phil., of Dec. 21, 1889, entitled "*Preliminary Observation on* (not 'a') *the Micro-organism of Texas Fever*," by Theobald Smith.

We need not notice this further, as the assertion is entirely without proof.

*To be continued.*

## Figures and Tables

**Fig. 1. f1:**
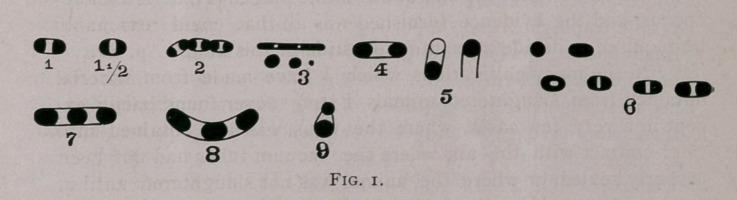


**Fig. 2. f2:**